# Exosome Secretion and Epithelial-Mesenchymal Transition in Ovarian Cancer Are Regulated by Phospholipase D

**DOI:** 10.3390/ijms232113286

**Published:** 2022-10-31

**Authors:** Hadil Onallah, Sheethal Thomas Mannully, Ben Davidson, Reuven Reich

**Affiliations:** 1 Institute of Drug Research, School of Pharmacy, Faculty of Medicine, The Hebrew University of Jerusalem, Jerusalem 91120, Israel; 2 Department of Pathology, Oslo University Hospital, Norwegian Radium Hospital, N-0310 Oslo, Norway; 3 Institute of Clinical Medicine, Faculty of Medicine, University of Oslo, N-0316 Oslo, Norway

**Keywords:** Phospholipase D, exosomes, ovarian cancer, epithelial-mesenchymal transition

## Abstract

Phospholipase D (PLD) isoenzymes participate in a variety of cellular functions that are mostly attributed to phosphatidic acid (PA) synthesis. Dysregulation of PLD regulates tumor progression and metastasis, yet little is known about the underlying mechanism. We previously reported on the expression and clinical role of the PLD isoenzymes PLD1 and PLD2 in tubo-ovarian high-grade serous carcinoma (HGSC). In the present study, we investigated the biological function of PLD1 and PLD2 using the OVCAR-3 and OVCAR-8 HGSC cell lines. KO cell lines for both PLDs were generated using CRISPR/CAS9 technology and assayed for exosome secretion, spheroid formation, migration, invasion and expression of molecules involved in epithelial-mesenchymal transition (EMT) and intracellular signaling. Significant differences between PLD1 and PLD2 KO cells and controls were observed for all the above parameters, supporting an important role for PLD in regulating migration, invasion, metastasis and EMT.

## 1. Introduction

Phospholipase D (PLD) hydrolyzes phosphatidylcholine to form phosphatidic acid (PA), one of the main membrane lipids [1,2]. Six different PLD isoforms have been identified, each isoform differently localized in the cell. PLD1 and PLD2 are the most studied isoforms, each displaying different functions and mechanisms. PLD1 is localized in intracellular compartments corresponding to granules or the Golgi apparatus, whereas PLD2 has been observed preferentially at the plasma membrane. It has been widely documented that PLD is associated with membrane vehicle trafficking and tumorigenesis [3,4,5]. Several studies have reported that PLD regulates tumorigenic pathways and is involved in the biosynthesis and secretion of exosomes, endogenous nanovesicles containing bioactive molecules that promote intercellular communication [2,3,6,7]. Inhibitors of PLD have been developed, aiming to achieve effective treatment and pharmacological inhibition of PLD in cancer invasion [8]. Depletion of PLD1 in melanoma and lung carcinoma tumor microenvironments results in reduced tumor burden [9]. In ovarian cancer (OC), the role of PLD has not been studied in depth. This malignancy is defined by metastasis in the form of malignant ascites and solid lesions within the abdominal cavity, creating a metastatic niche [10]. The formation of the tumor microenvironment is mediated by the secretion of exosomes from the primary tumor [11].

Our group has previously reported that both PLD1 and PLD2 are overexpressed in solid metastases and malignant effusions from patients with high-grade serous carcinoma (HGSC), the most common and clinically aggressive OC histotype [12]. The present study aimed to assess the effects of PLD1 and PLD2 on OC invasion, migration and exosome secretion. Our data highlight a novel potential role for PLD in mediating epithelial-mesenchymal transition (EMT) in this cancer.

## 2. Results

### 2.1. PLDs Are Involved in Exosome Secretion in OC Cell Lines

**Figure 1:**
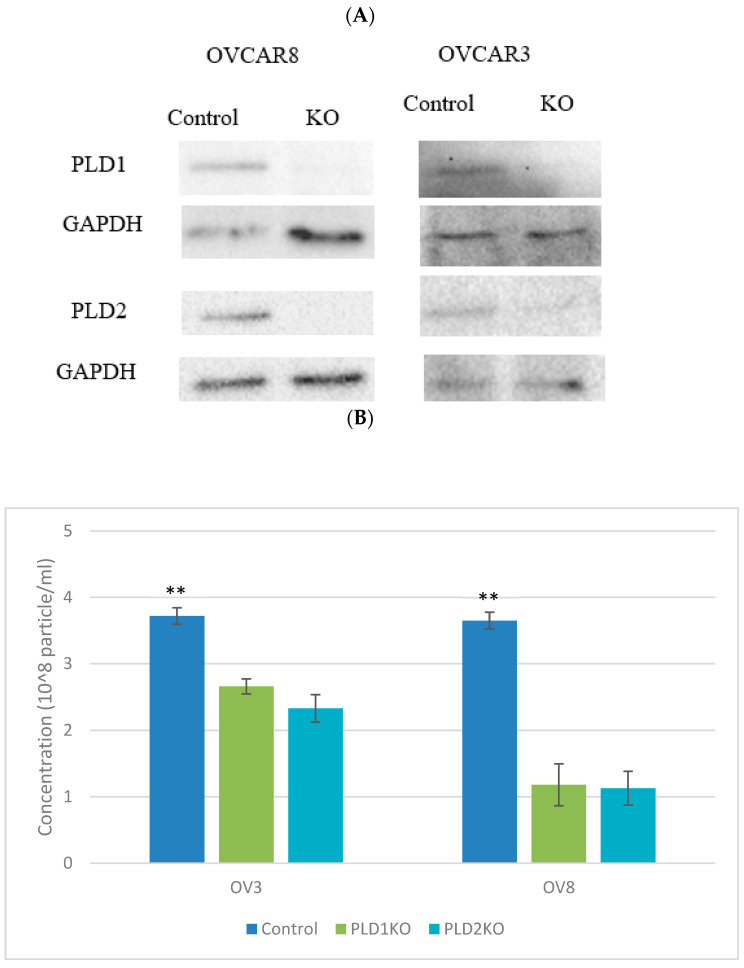

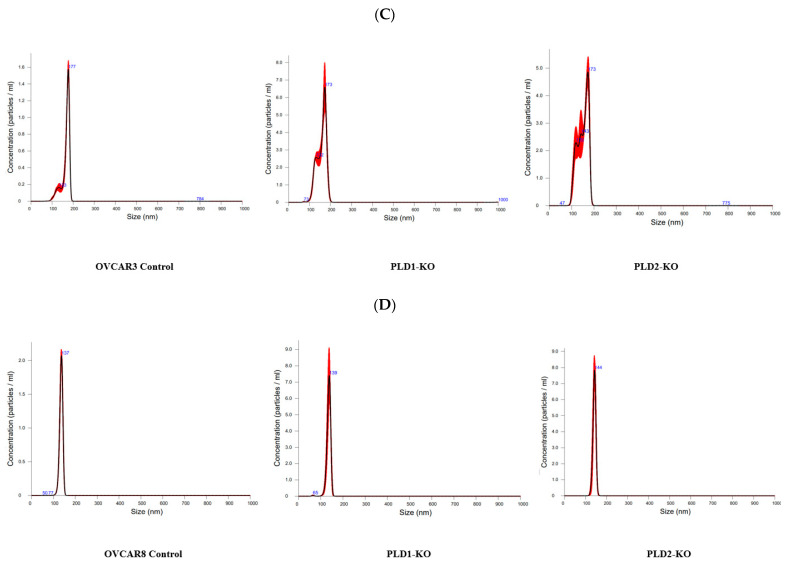
Exosome secretion in OVCAR3 and OVCAR8 control and KO cell lines. (**A**) Western blot demonstration of PLD1/2 CRISPR-Cas9 KO clones. (**B**) Exosomes particles concentration in OVCAR3 and OVCAR8 control and KO cells. Exosome secretion is reduced in PLD1/2-KO cells compared to control in both cell lines (** *p* < 0.01, n = 3). Error bars represent SEM. (**C**,**D**) Exosomes quantification using Nanosight NS3000 nanoparticle tracking analysis (NTA). Exosomes derived from all genotypes in the (**C**) OVCAR3 and (**D**) OVCAR8 cell lines were measured, PLD1-KO and PLD2-KO exosome secretion in both cell lines was significantly reduced.

PA is required for exosome production, and is produced by ARF6 and its effector PLD2 ^3^. We therefore designed two KO cell lines for both PLDs using the CRISPR/CAS9 method. When measured using Nanosight, exosome secretion was significantly reduced in both cell lines for both PLD1-KO and PLD2-KO compared to control cells (*p* < 0.01, Figure 1).

### 2.2. PLDs Suppression Affects Invasion and Migration in OC Cell Lines

**Figure 2:**
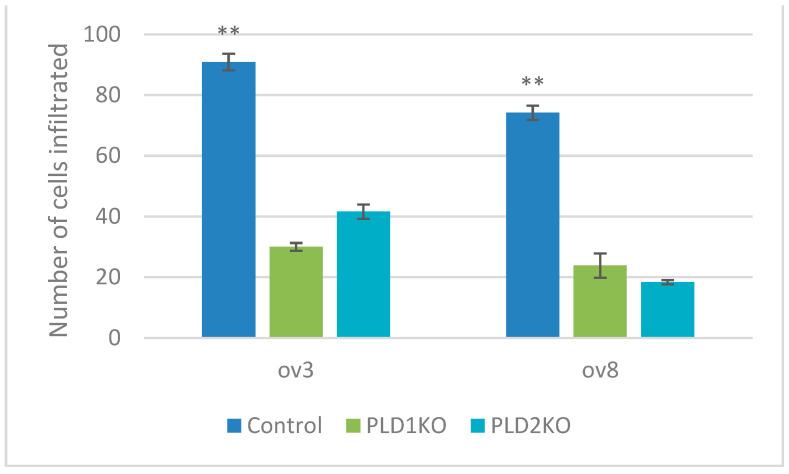
PLDs silencing affects invasion in OC cells. Transwell invasion assay to test the effect of PLDs on invasive activity. Average number of migrated cells is represented. Both KOs reduced invasion in both OVCAR3 and OVCAR8 cells (** *p* < 0.01, n = 6). Error bars represent SEM.

**Figure 3:**
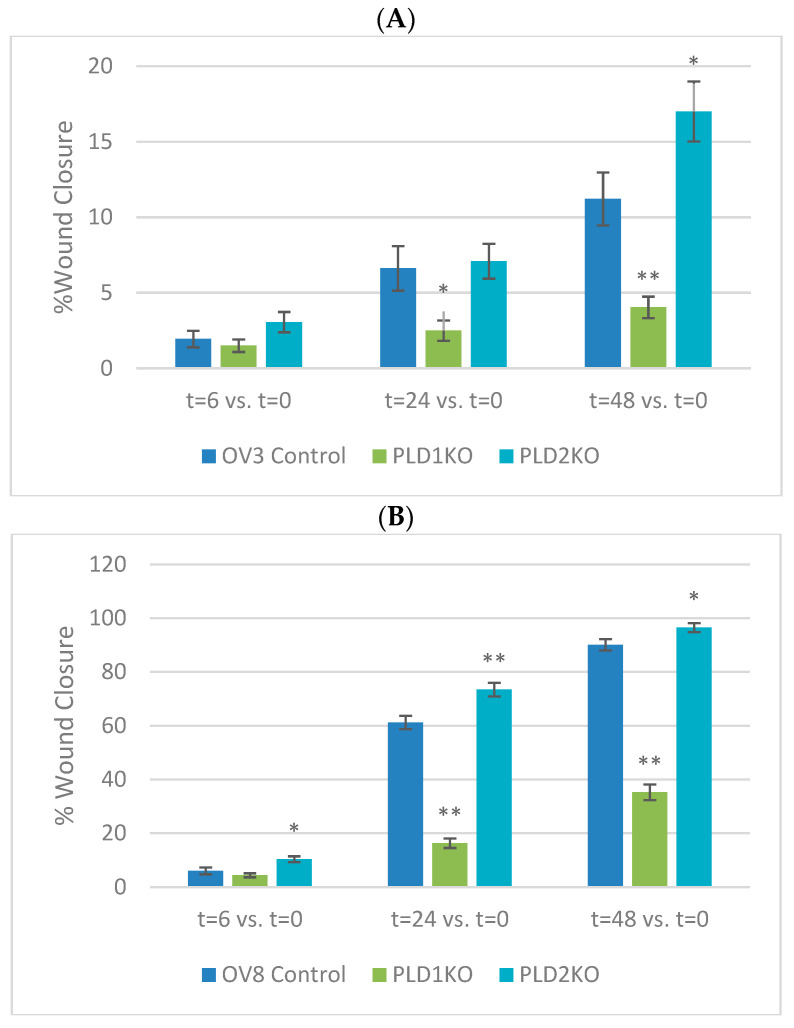

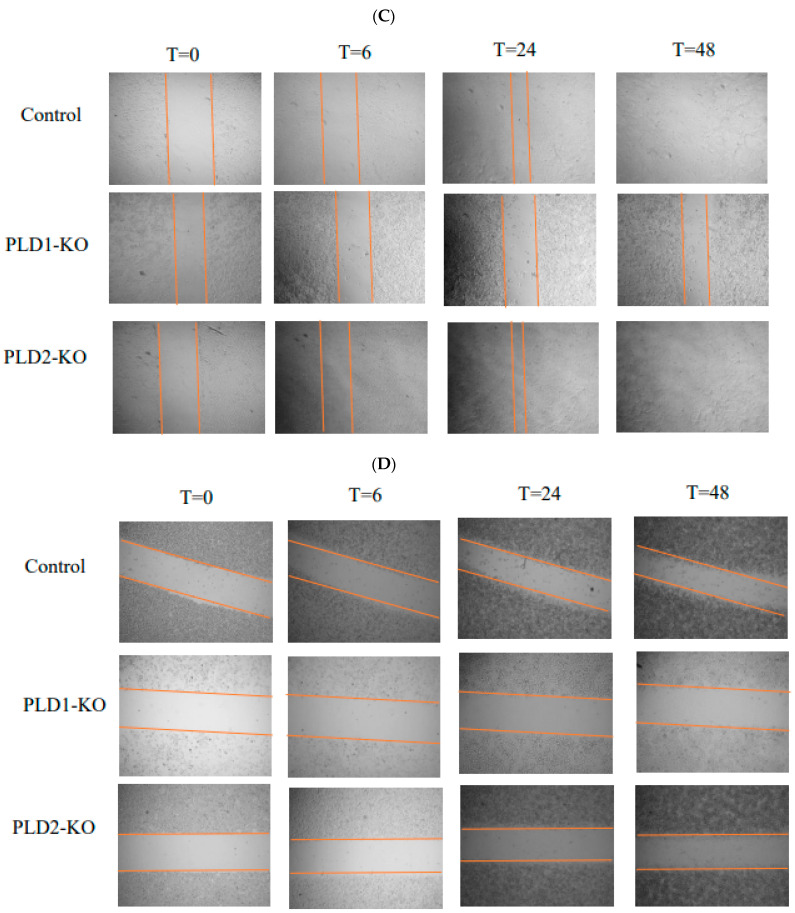
Cell migration (scratch wound healing assay). Wound healing assay in OVCAR3 and OVCAR8 cultured cells. Cells’ monolayers, cultured in 6-well plates, were injured with a sterile 10 μL pipette tip (original wound). (**A**,**B**) Graphs showing percentage of wound closure in both cell cultures in control and Knock out cells. PLD1-KO cells reduced motility whereas PLD2-KO cells increased cell motility compared to control cells (group size: n = 12 for each group). Values of percentage wound closure ± SEM; * *p* < 0.05, **, *p* < 0.01. Wound healing microscopy is shown to demonstrate cell migration in both cell cultures. (**C**) Representative images from in vitro scratch wound healing assays demonstrating that cell migration into the cell-free region is significantly reduced in PLD1-KO cells and increases in PLD2-KO cells. (**D**) Representative images from in vitro scratch wound healing assays demonstrating that cell migration into the cell-free region is significantly reduced in PLD1-KO cells and increases in PLD2-KO cells.

The transwell invasion assay was used to determine the invasive potential of PLD1/2-KO cells through the Matrigel layer [13]. PLD1 and PLD2 KOs had significantly reduced invasive ability in both cell lines compared to controls (*p* < 0.01, Figure 2). In contrast, statistical analysis at 24 and 48 h showed that the relative wound closure of PLD1-KO cells was significantly lower than control cells, whereas the relative wound closure of PLD2-KO cells was significantly higher than control cells (*p* < 0.05, Figure 3).

#### 2.2.1. PLDs Affect EMT in OC Cell Lines

The relative expression of E-cadherin, vimentin and fibronectin, three key molecules involved in EMT was analyzed.

**Figure 4:**
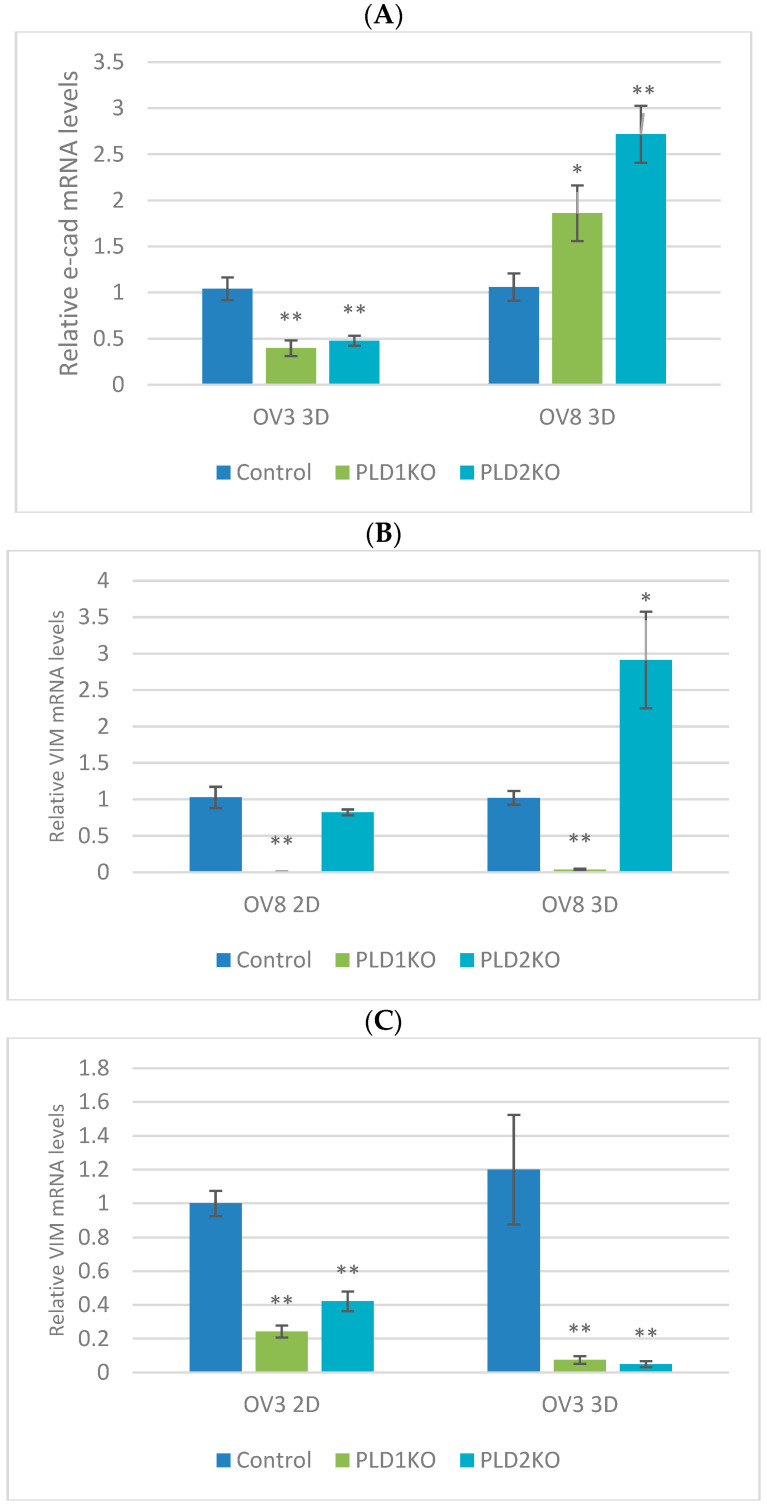

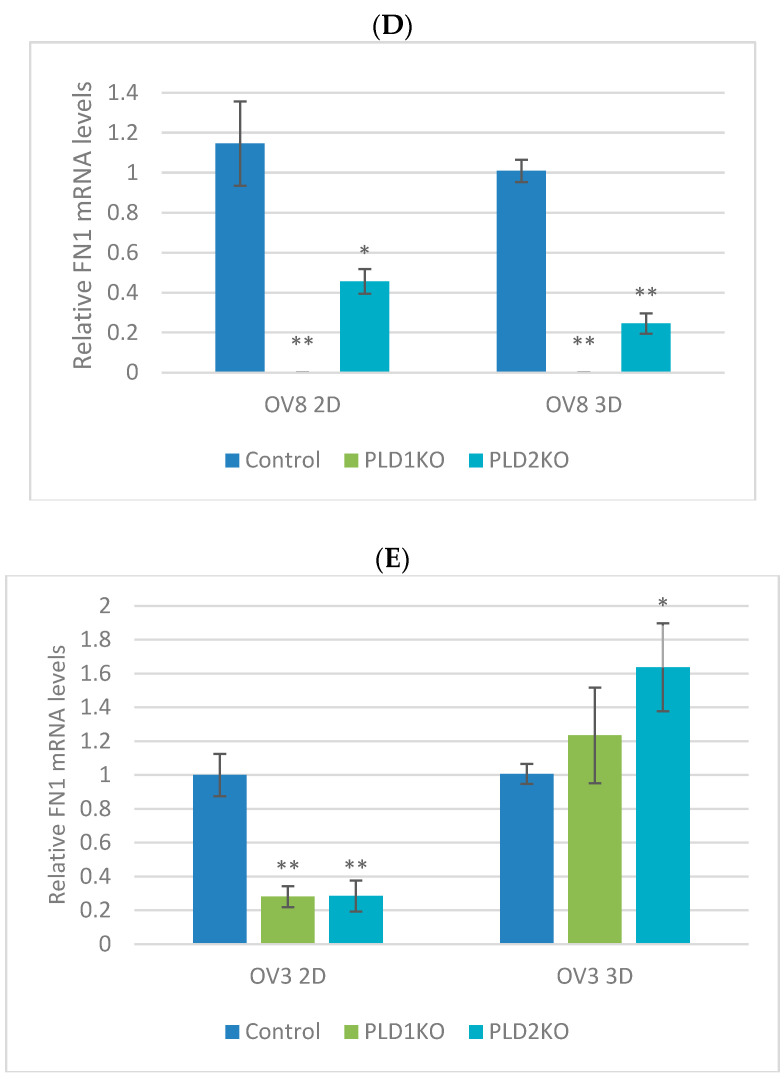
qPCR of vimentin, fibronectin and E-cadherin. Relative qPCR expression of E-cadherin (**A**), vimentin (**B**,**C**) and fibronectin (**D**,**E**) in OVCAR3 and OVCAR8 cells cultured in monolayer and spheroid form. * *p* < 0.05, ** *p* < 0.01.

As OVCAR8 is a cell line with a mesenchymal phenotype, both its monolayer and spheroid form did not express E-cadherin, whereas both PLD1/2KO cells expressed higher levels of E-cadherin in the spheroid form (Figure 4A). The relative mRNA levels of vimentin and fibronectin were reduced in PLD1/2 KO cells in a monolayer compared to controls, yet Vimentin mRNA levels were increased in spheroids (Figure 4B,D).

The OVCAR3 cell line has a less mesenchymal phenotype. Both Vimentin and fibronectin mRNA levels were decreased in PLD1/2 KO cells cultured as a monolayer compared to controls. In the spheroids, vimentin and E-cadherin levels were decreased, whereas fibronectin mRNA levels were slightly upregulated in PLD2-KO cells (Figure 4A,C,E).

#### 2.2.2. Suppression of PLDs Leads to Changes in POSTN and ANRIL mRNA Expression

**Figure 5:**
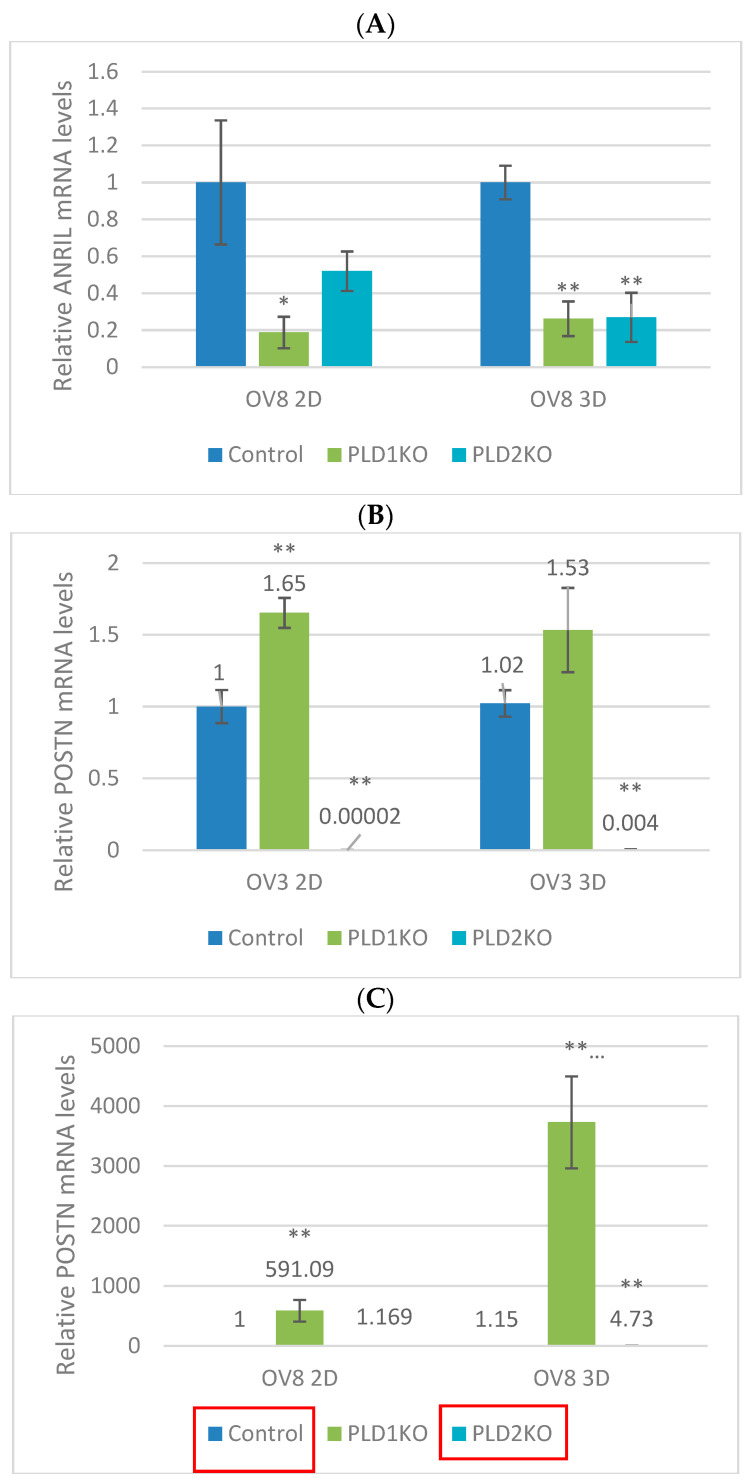
mRNA levels of ANRIL and POSTN. (**A**) ANRIL expression is reduced in PLD1-KO cells of OVCAR8 in both monolayer and spheroid form, whereas in PLD2-KO its reduced only in the spheroid form (* *p* < 0.05, ** *p* < 0.01). (**B**) POSTN mRNA levels are decreased in PLD2-KO of OVCAR3 in both forms (*p* < 0.01). (**C**) POSTN mRNA levels are induced in PLD1-KO of OVCAR8 in both forms (*p* < 0.01).

ANRIL, a long non-coding RNA (lncRNA), has been shown to be upregulated in OC [14,15]. ANRIL mRNA levels were significantly reduced in PLD1-KO cells in OVCAR8 cells compared to controls in both the monolayer and spheroid forms. PLD2-KO cells expressed decreased levels on ANRIL only in the spheroid form (*p* < 0.05, Figure 5A).

The mRNA levels of periostin, an EMT mediator, were elevated in PLD1-KO OVCAR3 cells cultured in monolayer, but were significantly reduced in PLD2-KO cells (*p* < 0.01, Figure 5B). In the OVCAR8 cell line, PLD1-KO expressed higher levels of POSTN in both forms (*p* < 0.01), yet in PLD2-KO cells it was induced only in the spheroids (*p* < 0.05, Figure 5C).

#### 2.2.3. PLD1 and PLD2 Have Opposite Effects on ERK Phosphorylation

**Figure 6:**
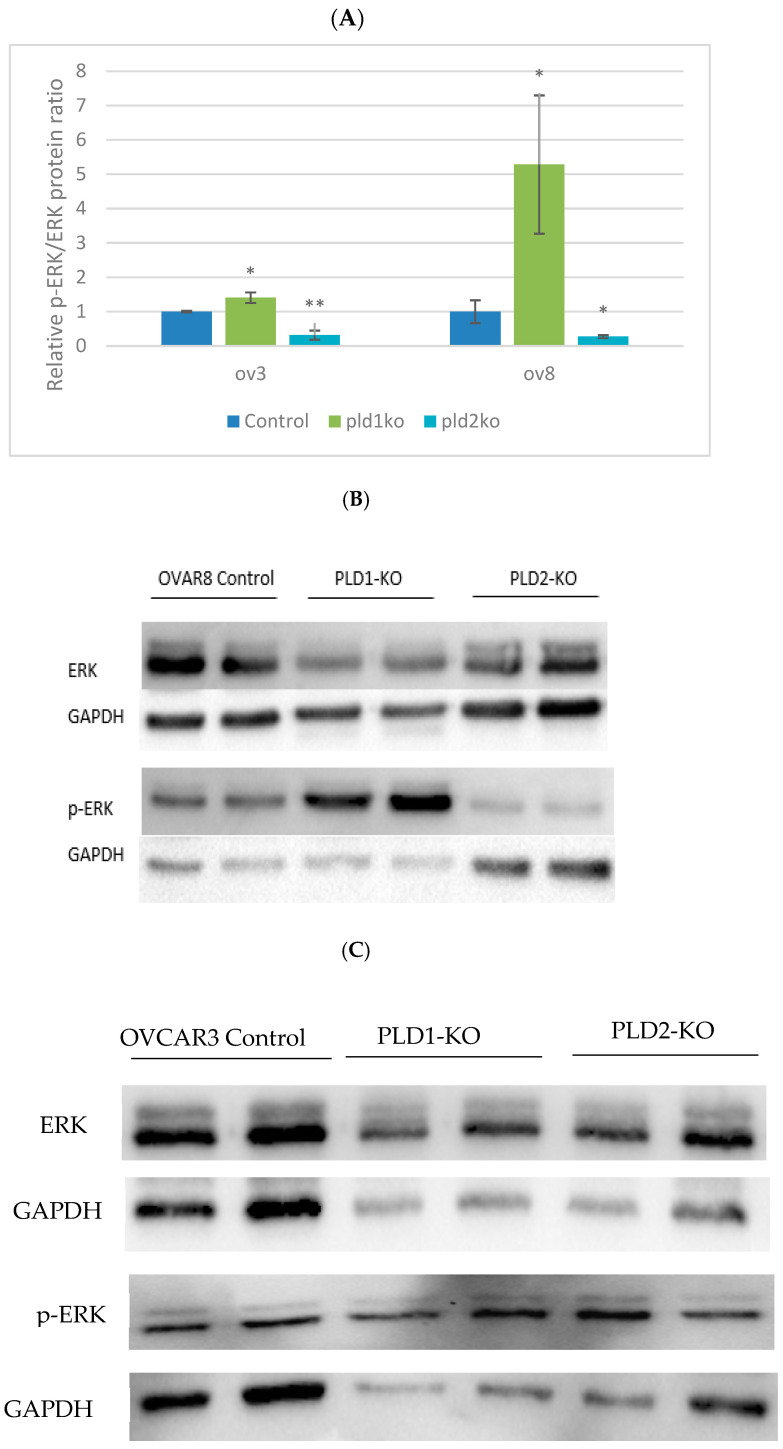
ERK expression is altered in PLDs KO cells. (**A**) Protein levels of p-ERK/ERK in OVCAR3 and OVCAR8 cell lines. p-ERK/ERK ratio is elevated in PLD1-KO (*p* < 0.05) and decreased in PLD2-KO cells (*p* < 0.05). Error bars represent SEM. (**B**,**C**) Representative western blot of both cell cultures is shown to demonstrate protein levels of ERK and p-ERK in control and KO cells (* *p* < 0.05, ** *p* < 0.01).

PLD1-KO cells expressed higher levels of p-ERK compared to control cells in both cell lines. On the other hand, lower levels were found in PLD2-KO cells compared with controls (*p* < 0.05, Figure 6), indicating that the two enzymes have opposite effects on ERK activity in OC cells.

#### 2.2.4. PLD1-KO Cells form Smaller Spheroids

**Figure 7:**
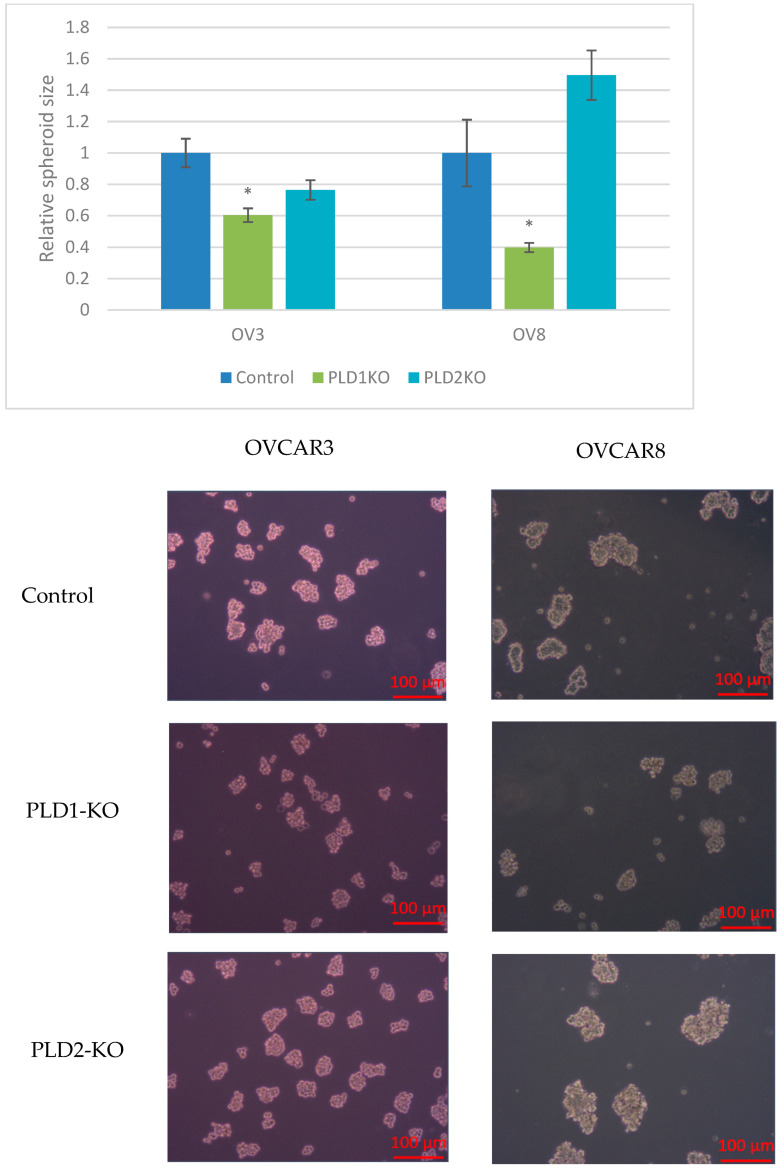
PLD1-KO cells generates significantly smaller spheroids. The perimeters of PLD1-KO spheroids were significantly smaller than those of controls in both cell lines (*p* < 0.05). Microscopy images display differences between control and KO cells (* *p* < 0.05).

We evaluated spheroid size in OVCAR3 and OVCAR8 cells. PLD1-KO cells generated significantly smaller spheroids in both cell lines compared to control cells (*p* < 0.05, Figure 7). No significant changes in spheroid size were detected for PLD2-KO cells.

## 3. Discussion

PLD enzymes and their altered activity have been implicated in various diseases, including cancer of various origin, yet their role in OC has not been fully elucidated. We previously reported that both PLD1 and PLD2 are overexpressed in solid metastases and malignant effusions compared with the ovarian tumor in HGSC. Additionally, higher PLD2 mRNA levels in malignant effusions were significantly related to less favorable responses to chemotherapy [12].

In the present study, PLD1 and PLD2 are shown to regulate exosome secretion in OC cells. Suppression of either PLD1 or PLD2 resulted in a significant decrease in exosome secretion. Ghossoub et al. reported on a novel pathway for ARF6 and its effector PLD2 in exosome biogenesis by controlling the budding of intra-luminal vesicles (ILVs) into multivesicular bodies (MVBs) [3]. For exosome budding, the signaling cascade of ARF6/PLD activates the ERK pathway. ARF6 recruits PLD to produce PA, which in turn activates ERK, affecting exosome budding [5]. Consistent with these findings, our results indicate that PLD2 inhibition results in a significant reduction in ERK. At present, there are no reports about PLD1-mediated exosome secretion, while our results indicate that PLD1 inhibition causes a major reduction in exosomes secretion despite increased ERK activity, indicating a different mechanism for exosome secretion [16,17,18].

An additional finding was elevated level of periostin mRNA in both PLD KOs. Periostin is a secreted protein that is enriched in exosomes and accumulates in ascites of OC patients. We hypothesize that due to suppression of exosome secretion in both PLD1 and PLD2 KOs, periostin was not secreted and accumulated in the cell.

PLD1 and PLD2 KO inhibited cell invasion in vitro, suggesting that both isoenzymes promote invasion. However, opposing results were seen with respect to migration, suggesting that PLD1 positively regulates cell migration while PLD2 suppresses it.

One of the main features of advanced-stage OC is the formation of spheroids within the peritoneal cavity [19]. PLD1 KO cells had reduced spheroid size in the OVCAR8 cell line that forms compact spheroids, and also in the OVCAR3 cell line that forms loose aggregates. The different spheroid form in these two cell lines is attributed to different expression of EMT markers [20]. In the present study, we found that both PLD enzymes regulate EMT. KO of both PLD isoenzymes suppresses vimentin and fibronectin levels when cells are cultured in monolayer, and increases the expression of E-cadherin in the spheroid form. Fite K. et al. reported that PLD-targeting microRNAs are regulated by EMT in breast cancer [21]. Ganesan R. et al. reported that PLD2 in human breast tumors is regulated by the Slug and Snail transcription factors that are highly implicated in enhancing the invasiveness of cancer cells by promoting EMT [22].

**Figure 8:**
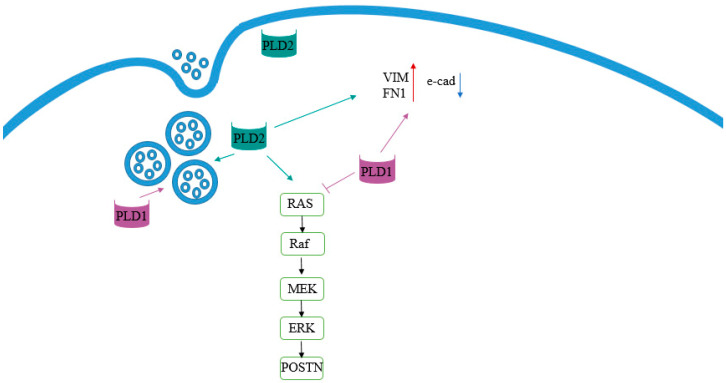
Suggested model for PLD signaling pathway.

There has been an emerging body of work supporting the association between altered levels of lncRNAs and OC in recent years, suggesting a role for these molecules in tumor development and progression. ANRIL, encoded in the chromosome 9p21 region, has been implicated in cancer progression through promotion of metastasis and EMT, and its expression has been reported in OC [23]. In the latter cancer, elevated ANRIL levels have been correlated with advanced FIGO stage [15,23]. Qiu et al. reported that ANRIL regulates serous OC migration and invasion by regulating fibronectin and the Met proto-oncogene, and detected increased levels of E-cadherin following silencing of ANRIL [24]. These findings are well in agreement with our finding of reduced level of both ANRIL and fibronectin in PLD knockouts. Moreover, suppression of PLD enzymes resulted in an increased level of E-cadherin in OVCAR8 spheroids, together with a significant reduction in ANRIL (Figure 8). Animal studies need to be performed in order to reproduce our results using an in vivo approach, whose absence constitutes the major limitation of the present study.

## 4. Materials and Methods

### 4.1. Cell Lines and Reagents

The OVCAR3 and OVCAR8 OC cell lines, both widely regarded to originate from HGSC, were obtained from the American Type Culture Collection (ATCC) and cultured according to the manufacturer’s instructions. OVCAR3 cells were cultured in DMEM, OVCAR8 in RPMI (Biological Industries, Beit-Haemek, Israel). The medium was supplemented with 1% L-glutamine, 1% sodium pyruvate, 1% vitamin solution, 1% non-essential amino acids (Biological Industries) and 10% fetal calf serum (Sigma-Aldrich, St. Louis, MO, USA). Cells were grown in a humidified atmosphere of 95% air and 5% CO2. In cells from which exosomes were extracted, the medium used was EXCELL^®^ Advanced™ CHO Fed-batch Medium (Sigma-Aldrich). OVCAR3 and OVCAR8 OC spheroids were formed by constant shaking of 400,000 cells per well in 6-well plates for 48 h on a vertical shaker.

### 4.2. qRT-PCR

cDNA was transcribed of 1000 ng total RNA. qRT-PCR was carried out using the PerfeCTa SYBR^®^ Green FastMix qPCR reagent (Quantabio, Beverly, MA, USA) according to the manufacturer’s protocol. Specificity was confirmed by appropriate melting curves. mRNA levels were established by calculating the ratio between the target molecule and the reference gene RPLP0. Primer sequences are listed in Supplementary Table S1.

### 4.3. Immunoblotting

Cells and exosomes were lysed in a RIPA buffer containing 1% NP-40, 20 mM Tris-HCl (pH 7.5), 137 mM NaCl, 0.5 mM EDTA (Mallinckrodt Baker Inc., Philipsburg, NJ, USA), 10% glycerol (Frutarom LTD, Haifa, Israel), 1% protease inhibitor cocktail (Sigma-Aldrich) and 0.1% SDS (Biological Industries). After centrifugation, protein content was quantified using the Bradford assay, and 25 μg of protein from each specimen were heat-denaturated in a Laemmli sample buffer and fractionated in 10% gels by SDS–PAGE, electro-transferred to nitrocellulose membrane for blotting with antibodies. In order to block nonspecific binding, membranes were incubated for 1 h in 5% low-fat milk dissolved in TBST. Membranes containing proteins originating from exosomes did not undergo blocking as per the manufacturer’s instruction for CD81 antibody. Membranes were then incubated with the following antibodies: ERK monoclonal antibody (#4695, Cell Signaling Biotechnology, Danvers, MA, USA), p-ERK monoclonal antibody (#4377, Cell Signaling Biotechnology) and CD81 polyclonal antibody (PA5-13582, Thermo Scientific Inc., Waltham, MA, USA). GAPDH (14C10; Cell Signaling Biotechnology) was used as loading control. Blots were developed in Bio-Rad ChemiDoc™ XR and analyzed using Image Lab™ software.

### 4.4. Generation of Knockout Cells Using CRISPR/Cas9

CRISPR/Cas9-mediated editing was performed as previously described by the Zhang Lab [25]. Transfections were done with the Avalanche^®^-Everyday Transfection Reagent (EZ Biosystems, College Park, MD, USA). Single-cell clones were screened by immunoblotting for detection of a knockout. Specific single guide RNA (sgRNA) inserts were designed with the help of Zhang Lab scoring (http://crispr.mit.edu/) accessed on 23 August 2016 and were targeted at the beginning of the 5′ of each gene. gRNA sequences used for human PLD1: 5′-CACCGGAACTCCACTTTGAGGGAG-3′; 5′-AAACCTCCCTCAAAGTGGAGTTCC-3′, and human PLD2: 5′-CACCGCTCCAGATGGAGTCCGATG-3′; 5′-AAACCATCGGACTCCATCTGGAGC-3′.

### 4.5. Exosome Extraction and Quantitation

3 × 10^6^ cells were seeded and cultured in serum-free medium for 48 h. Conditioned media were collected and centrifuged at 2000× *g* for 10 min to remove cells and debris, filtered with a 0.2 μM PVDF filter (Merck Millipore, Burlington, MA, USA) and then concentrated with 100K MWCO Vivaspin 20 (Sartorius, Göttingen, Germany). Exosomes were further extracted from the supernatant according to the ExoQuick-TC manual. The resulting pellet-containing exosomes were re-suspended in 100 μL PBS and analyzed for protein concentration by the Bradford assay. Exosomes were quantified using Nanosight Nanoparticle Tracking Analysis (QuantumDesign, San Diego, CA, USA). Immunoblot for control cells was performed for the presence of CD81 (Supplementary Figure S1).

### 4.6. Wound Healing Assay

OVCAR3 and OVCAR8 with PLD1 and PLD2 KOs and control cells were seeded to confluence in 6-well plates. Each well was scratched twice with a sterile tip and imaged at t = 0, t = 6, t = 24 and t = 48 h. The closure of the wound was analyzed by T-scratch software.

### 4.7. Invasion Assay-Boyden Chamber

Cells of all genotypes (n = 50,000) were seeded on Matrigel-coated filters in Boyden chambers, whereas a chemoattractant (conditioned medium from the 3T3 fibroblast cell line) was placed on the opposite side. Six hours after incubation at 37 °C, filters were removed and the presence of invading cells was determined by staining and quantification under an inverted microscope.

### 4.8. Spheroid Size Evaluation

Spheroid size was evaluated by measuring spheroid diameter using Image J software (NIH, Bethesda, MD, USA). Results were expressed as relative mean pixel measure ± S.E.M. Pictures for spheroid size evaluation were taken with an Axiocam 105 color microscope camera magnification ×10 (Zeiss, Oberkochen, Germany).

### 4.9. Statistical Analysis

Student’s *t*-test was employed; probability of *p* < 0.05 was considered statistically significant. High and low mRNA and protein expression was defined relative to expression in control cells in each experiment.

## 5. Conclusions

Altogether, our data suggest that PLD activity could serve a pivotal role in the progression and exosome secretion of OC. Targeting of PLD isoenzymes or their regulators may potentially have a therapeutic potential in this disease.

## Supplementary Materials

The following supporting information can be downloaded at: https://www.mdpi.com/article/10.3390/ijms232113286/s1.
