# Supplementary material for: Exosome Secretion and Epithelial-Mesenchymal Transition in Ovarian Cancer Are Regulated by Phospholipase D
Source: Int J Mol Sci. 2022 Oct 31;23(21):13286. doi: 10.3390/ijms232113286 (PMC9658871; doi:10.3390/ijms232113286)

Supplementary data

Table S1: Primer sequences

Gene		Primer sequence (5' → 3')
E-cadherin	Forward	GCCATCGCTTACACCATCCT
	Reverse	GGCACCTGACCCTTGTACGT
Vimentin	Forward	TCTGGATTCACTCCCTCTGGTT
	Reverse	CGTGATGCTGAGAAGTTTCGTT
Fibronectin	Forward	GCGAGAGTGCCCCTACTACA
	Reverse	GTTGGTGAATCGCAGGTCA
Periostin	Forward	GTCTTTGAGACGCTGGAAGG
	Reverse	AGATCCGTGAAGGTGGTTTG
ANRIL	Forward	TGCTCTATCCGCCAATCAGG
	Reverse	GGGCCTCAGTGGCACATACC
RPLP0	Forward	CCAACTACTTCCTTAAGATCATCCAACTA
	Reverse	ACATGCGGATCTGCTGCA

Figure S1: CD81 and Ponceau expression in exosomes extracted from OVCAR3 and OVCAR8 control cells

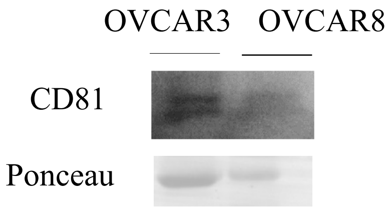

Supplement: Supplementary file 1 [file ijms-23-13286-s001.zip › ijms-1927596-supplementary.pdf]
